# The spatio-temporal dynamics of deviance and target detection in the passive and active auditory oddball paradigm: a sLORETA study

**DOI:** 10.1186/s12868-018-0422-3

**Published:** 2018-04-19

**Authors:** Christoph Justen, Cornelia Herbert

**Affiliations:** 1 University of Tuebingen, Tuebingen, Germany; 2 Institute of Psychology and Education, Applied Emotion and Motivation Research, University of Ulm, Ulm, Germany

**Keywords:** EEG, Source localization, N1, Mismatch negativity, MMN, P3, Attention, Salience, Attention networks

## Abstract

**Background:**

Numerous studies have investigated the neural underpinnings of passive and active deviance and target detection in the well-known auditory *oddball paradigm* by means of event-related potentials (ERPs) or functional magnetic resonance imaging (fMRI). The present auditory oddball study investigates the spatio-temporal dynamics of passive versus active deviance and target detection by analyzing amplitude modulations of early and late ERPs while at the same time exploring the neural sources underling this modulation with standardized low-resolution brain electromagnetic tomography (sLORETA) .

**Methods:**

A 64-channel EEG was recorded from twelve healthy right-handed participants while listening to ‘standards’ and ‘deviants’ (500 vs. 1000 Hz pure tones) during a passive (block 1) and an active (block 2) listening condition. During passive listening, participants had to simply listen to the tones. During active listening they had to attend and press a key in response to the deviant tones.

**Results:**

Passive and active listening elicited an N1 component, a mismatch negativity (MMN) as difference potential (whose amplitudes were temporally overlapping with the N1) and a P3 component. N1/MMN and P3 amplitudes were significantly more pronounced for deviants as compared to standards during both listening conditions. Active listening augmented P3 modulation to deviants significantly compared to passive listening, whereas deviance detection as indexed by N1/MMN modulation was unaffected by the task. During passive listening, sLORETA contrasts (deviants > standards) revealed significant activations in the right superior temporal gyrus (STG) and the lingual gyri bilaterally (N1/MMN) as well as in the left and right insulae (P3). During active listening, significant activations were found for the N1/MMN in the right inferior parietal lobule (IPL) and for the P3 in multiple cortical regions (e.g., precuneus).

**Discussion:**

The results provide evidence for the hypothesis that passive as well as active deviance and target detection elicit cortical activations in spatially distributed brain regions and neural networks including the ventral attention network (VAN), dorsal attention network (DAN) and salience network (SN). Based on the temporal activation of the neural sources underlying ERP modulations, a neurophysiological model of passive and active deviance and target detection is proposed which can be tested in future studies.

## Background

Rapid orientation of selective attention towards changes in the acoustic environment is essential for successful interaction with our environment. Remarkably, the deviance of a perceived stimulus determines to what extent attention is captured by this stimulus. In other words, the more deviant a stimulus is, the more attention is allocated towards this particular stimulus [1]. In the auditory domain, the essential ability of deviance detection has been studied mostly in the so-called *oddball paradigm* [2]. In this experimental paradigm two auditory stimuli (e.g., pure tones) are presented as targets (*deviants*) or as non-targets (*standards*). Deviants are usually embedded in a continuous stream of standards and differ from standards in at least one perceptual dimension (for instance, a difference in frequency, pitch or loudness). This difference in stimulus presentation frequency and in physical stimulus properties between deviants and standards seems sufficient to prioritize processing of deviants over standards during passive and hence, involuntary (*bottom*-*up*) stimulus processing. In addition, detection of deviant stimuli may benefit from active, i.e., voluntary (*top*-*down*) controlled stimulus processing, for instance, when deviants are actively attended by the participants as targets for task-related voluntary discrimination of deviants and standards [3].

Due to its simplicity, the auditory oddball paradigm constitutes an ideal research paradigm for cognitive neuroscience to investigate the neural mechanisms of auditory deviance and target detection during passive and active listening conditions. So far, several event-related potential (ERP) and functional magnetic resonance imaging (fMRI) studies have been conducted to determine on the one hand the time course and on the other hand the brain regions underlying auditory deviance and target detection in this paradigm (for meta-analytic research e.g., [4]). Effects have been investigated during task conditions of passive or active listening (for ERP studies see e.g., [5–8]; for fMRI see e.g., [9–13]). In general, this body of research provided important insight into auditory processing under unattended (passive) and attended (active) task conditions while at the same time raising questions about the temporal activation of specific brain regions and brain networks involved in auditory deviance and target processing during passive versus active listening conditions.

According to influential theoretical models of attention allocation proposed by Corbetta and Shulman [14] and data from a recent meta-analysis based on functional imaging studies [4], processing of deviant stimuli (in contrast to standard stimuli) is associated with bottom-up as well as top-down stimulus processing and activation of two distinct fronto-parietal networks, namely the ‘dorsal attention network’ (DAN) and the ‘ventral attention network’ (VAN) [4, 14, 15]. The DAN comprises the superior parietal lobule/precuneus (SPL; Brodmann area (BA7)), the intraparietal sulcus (IPS; BA 6/7), parts of the middle temporal cortex (BA 21) and the inferior frontal junction (IFJ; BA 6/9/44). The VAN comprises the temporoparietal junction (TPJ; BAs 39/40), the supramarginal gyrus (SMG; BA 39), the superior temporal gyrus (STG; BAs 22/41/42), the frontal operculum (FO; BA 44/45/47), the inferior frontal gyrus (IFG; BAs 44/45/47), the anterior cingulate cortex (ACC; BAs 24/32/33) and the anterior insula (AI, BA 13) [4, 14, 15]. According to recent meta-analytic research, putting together the results of 75 neuroimaging studies using either auditory or visual oddball paradigms, processing of deviants versus standards activates the DAN and the VAN differently during passive versus active stimulus processing [4]. Parts of the DAN seem to be activated during the processing of deviants and standards, and specifically its frontal parts seem to be involved in voluntary target detection in line with the idea of the dorsolateral prefrontal cortex (DLPFC; BAs 8/9/10/46) playing a central role in top-down selective attentional control [4, 16]. In contrast to the DAN, the VAN seems to be exclusively involved in the detection of deviant stimuli and brain regions belonging to the VAN seem to be more active when deviants are voluntarily attended as compared to conditions in which they are not attended (e.g., during passive listening). The VAN is therefore supposed to enable ‘attentional shifting’ to deviant stimuli, probably to initiate an appropriate behavioral response when deviants are important for the task [17].

However, this raises questions about which brain networks and brain regions might be involved in auditory deviance processing when there is no task at hand. Theoretically, some brain structures of the VAN such as the insula and ACC but also brain regions of the DAN (e.g., precuneus) form part of the ‘salience network’ (SN) [18, 19]. Amongst these brain regions in particular the insular cortex is considered to be involved in bottom-up detection of salient events and the selection of these for additional processing [20]. In addition, it has been shown that in the oddball paradigm, attentional processing (e.g., attention orientation) to auditory stimuli can activate visual processing regions (e.g., lateral and medial occipital areas) and hence, processing regions typically engaged in object recognition and in the perception of visual objects [21, 22] in tasks requiring spatial attention (e.g., see [23]). Thus, specific brain regions in the visual cortex (such as the lateral occipital cortex (LOC) or the lingual gyrus (LG) in the medial visual cortex) may also be activated during attentive processing of acoustic stimuli [24]. However, whether the aforementioned visual processing areas are also activated during task conditions of passive and thus, unattended listening to deviant and standard pure tones in the oddball paradigm needs to be investigated further [25–27].

So far, the above outlined assumptions about the activation of the DAN and VAN during auditory processing have been derived mainly from meta-analytic research [4] including functional neuroimaging studies. Thus, it has not been investigated whether activation of the DAN and VAN could be modeled by source imaging techniques that rely on EEG activity such as sLORETA. Crucially, exploring the time course of auditory deviance and target detection through electroencephalography (EEG) and event-related brain potentials (ERPs) and simultaneous targeting the brain regions of the VAN and DAN by means of sLORETA could be especially fruitful in situations where fMRI is not available or too costly. Moreover, due to its high temporal resolution in the millisecond time-range, EEG and EEG based source imaging offers the possibility to investigate the time course of deviance and target detection in the auditory oddball paradigm (e.g., be investigating modulation of ERPs) while at the same time the neural sources underlying ERP modulation can be estimated within the same temporal resolution of milliseconds. Akin to functional imaging, in EEG-ERP source imaging studies, effects can be investigated during passive listening conditions, during which intentional discrimination between deviants and standards via explicit instructions is not required. In addition, investigation of effects associated with active listening conditions (e.g., explicit instruction to attend to deviant stimuli) is also possible, thus allowing direct comparisons of ERPs, their neural sources and their modulation during deviance and target detection in a within-subject design including task conditions of passive and active listening.

Regarding the time course of auditory deviance and target detection, ERP components most consistently elicited during the auditory oddball paradigm are the N1, the N2a or auditory mismatch negativity (N2a/MMN; e.g., see [28]), and the late P3 (e.g., see [29–31]).Whereas the N1 and MMN are brain potentials whose amplitudes are significantly influenced by differences in physical stimulus properties, the magnitude of the P3 amplitude is significantly influenced by cognitive and task demands (e.g., see [32]). The N1 and the MMN have been found during passive listening as well as during active processing of deviant stimuli [33]. In contrast to the N1, the MMN is computed by subtracting the averaged ERP waveform of standard stimuli from the averaged ERP waveform of deviant stimuli. The resulting negative deflection is peaking between 100 and 250 ms after stimulus onset [34]; amplitudes of the MMN being often more pronounced at fronto-central electrode sites [35]. Regarding its latency, the MMN can overlap with the auditory N1 component (e.g., see [36]) peaking between 80 and 120 ms post-stimulus [37], especially if deviant and standard stimuli are clearly perceptually distinct from each other. The auditory MMN has been proposed to indicate mostly pre-attentive sensory stimulus discrimination [38] as well as automatic, and thus involuntary auditory change detection [39, 40]. Hence, the modulation of the MMN is assumed to be driven by involuntary mechanisms of the brain’s sensory processing system matching the incoming stimulus to its internally stored representation (or template). This matching is considered to occur “unconsciously” and temporally prior to stimulus categorization [41].

Amplitudes of the P3, temporally following early brain potentials such as the N1 and N2a/MMN, are most pronounced between 300 and 450 ms after stimulus-onset at central-parietal as well as parietal electrode sites [30]. Previous auditory oddball studies suggest that the P3 is elicited only by sufficiently deviant stimuli [42] with P3 amplitudes being larger in response to voluntarily attended than unattended deviant stimuli (e.g., see [5]). Accordingly, the P3 is thought to reflect voluntary switch of attention [39, 40, 43] and in depth-processing of a stimulus signaling stimulus evaluation based on memory [44] and context updating [29, 45].

Taken together, one might expect N1, N2a/MMN and P3 modulation to be differentially sensitive to task effects. Moreover, one could speculate that different neural sources may underlie N1, MMN and P3 modulation during deviance and target detection in passive and active listening conditions. Compared to the huge amount of EEG–ERP studies or fMRI studies investigating either the time course or the brain regions involved in auditory deviant and target detection only few studies investigated both, the time course and the neural sources of auditory deviance and target detection in the oddball paradigm, for instance with combined EEG–fMRI methodology [13]). Although combining fMRI and EEG/ERP methodology is beneficial, localization of neural generators of ERP components might still be affected by the relatively poor temporal resolution of conventional functional neuroimaging techniques [46]. To this end, (standardized) low-resolution brain electromagnetic tomography (LORETA and sLORETA, respectively) have been developed to make assumptions about the location of neural generators of brain electrical activity derived from multi-channel EEG recordings with high temporal resolution [47–49]. The validity of (s)LORETA has been confirmed in a number of combined fMRI-EEG studies (e.g., see [48]). More specifically, Mulert et al. compared brain activations in an active auditory oddball paradigm as measured by fMRI with brain activations derived from EEG and LORETA [50]. The comparison between fMRI and LORETA activation patterns contrasting deviants versus standards during active and hence, attended processing of deviants versus standards showed concurrent activations in brain regions associated with the VAN, namely the TPJ (BAs 39/40), the supplementary motor area (SMA; BA 6), the ACC (BAs 24/32/33), the middle frontal gyrus (MFG; BA 46) and the anterior insula (AI, BA 13). Interestingly, neural generators of the P3 partially overlapping with the VAN, including TPJ (BAs 39/40), DLPFC (BAs 8/9/10/46), ACC (BAs 24/32/33) and parts of the parietal and temporal cortices could be already confirmed in recent EEG-sLORETA studies (e.g., see [50–52]). Thus, source localization with sLORETA can be a means to successfully estimate neural sources underlying target detection in the oddball paradigm without losing information about the time course.

### Aim and objectives of the present study

Building upon the previous findings outlined above, the aims of the present auditory oddball study were to methodologically combine EEG–ERP methodology with sLORETA in the oddball task in order to (a) investigate the time course of auditory deviant and target detection by studying ERP modulation in active as well as in passive listening conditions and (b) examine the temporal activation of brain regions contributing to the observed ERP modulation patterns in the two different listening conditions. Thus, by taking advantage of the high temporal resolution of the sLORETA source imaging technique the present study allows to explore which brain regions belonging to the hypothesized brain networks (i.e., DAN, VAN and SN) might contribute to the modulation of early and late ERP components elicited by deviant and standard pure tones during active and passive listening. In line with previous research and the assumptions outlined above the present study aimed at testing the following hypotheses and open questions regarding the temporal activation of specific brain regions during auditory deviant and target detection: firstly, we were interested in whether passive listening of deviant compared to standard stimuli will be associated with activations in brain regions belonging to the VAN or the SN, indicating involuntary attentional orientation towards deviant stimuli when no behavioral response is required. Secondly, we were interested in whether during passive listening brain regions belonging to the VAN and, or the SN will contribute as neural sources to automatic deviant detection (MMN) or to later processing stages of deviance processing as indicated by modulation of the P3. Thirdly, during the attended oddball paradigm, activations in brain regions associated with the VAN and the DAN could be expected in the time window of the MMN and the P3 component indicating (1) involuntary attentional orientation towards deviant stimuli and (2) voluntary modulation of attention in order to maintain attentional resources for responding behaviorally to deviant stimuli. Finally, previous fMRI studies found activation in the visual cortex during passive auditory processing in the oddball paradigm. Thus, cortical activations in lateral/medial occipital areas could also be expected to occur in the present study during passive listening and activation of these brain regions might be associated with the modulation of early and late ERPs.

## Methods

### Participants

Twelve right-handed university students (7 females, 5 males) aged between 19 and 26 years (*M* = 21.3 years, *SD* = 2.15) participated in the present study. All participants were in good health and reported no psychological or hearing disorders. The experiment (including EEG recordings) was conducted at the Institute of Psychology of the German Sport University Cologne, Germany and was part of a larger project of the authors (see funding sources, and [53]). The experimental protocol complied with the Declaration of Helsinki and was approved by the local ethics committee of the German Sport University Cologne, Germany. All participants gave written informed consent prior to the start of the experiment and received a momentary compensation for their participation.

### General procedure

Prior to the start of the EEG session, participants had been seated in a comfortable chair and were informed about the general procedures of the experiment including EEG recordings. The experiment consisted of a passive and active auditory oddball paradigm; the passive condition (block 1) being always followed by the active condition (block 2). Block order was kept constant across participants as not to confound passive with active processing due to possible carry over effects. Particularly, the induction of an overt (behavioral) response to deviant pure tones during the active paradigm and hence active orientation of attention towards the deviant stimuli may have influenced deviance processing during passive listening if the active paradigm had been presented first (cf., [5]). Auditory stimuli were presented at constant sound pressure level of about 75 dB/SPL using Shure SHR440 on-ear headphones (Shure, Niles, IL, USA). During the *passiv*e oddball paradigm (block 1), participants were instructed to listen passively to the presented auditory stimuli. Accordingly, no behavioral response was required. For the active oddball paradigm (block 2), participants were told to respond to the deviant pure tones as quickly as possible by pressing the space bar on a keyboard with their right index finger. To avoid horizontal eye movements (saccades) while listening, participants were instructed to fixate their view on a fixation cross presented on the video screen. Additionally, participants were told to keep their eyes open during stimulus presentations (block 1 and block 2, respectively).

### Passive and active tone oddball paradigm

The oddball paradigm consisted of two auditory stimuli, a low (500 Hz) pure tone which was presented as “standard” and a high (1000 Hz) pure tone which was presented as “deviant” stimulus (cf., [54]). Thus, both pure tones differed only in the frequency domain and both were perceptually sufficiently different from each other to be clearly recognized as standard and deviant during stimulus presentation. Both auditory stimuli had durations of 50 ms (including a 5 ms fade-in/out time). The experimental paradigm consisted of in total of 400 trials (325 standard trials and 75 deviant trials, respectively) with a fixed inter-stimulus-interval (ISI) of 950 ms duration. Stimulus presentation and recording of responses were controlled by the Inquisit 4.0 software package (Millisecond Software, Seattle, WA, USA). The experimental script was downloaded from the official Inquisit website ([55]).

### EEG recordings

Continuous EEG data (sampling frequency: 2.048 Hz) were recorded from 64 Ag/AgCl sintered electrode using standardized EEG recording sites (Fp1, Fpz, Fp2, AF7, AF3, AF4, AF8, F7, F5, F3, F1, Fz, F2, F4, F6, F8, FT7, FC5, FC3, FC1, FCz, FC2, FC4, FC6, FT8, T7, C5, C3, C1, Cz, C2, C4, C6, T8, TP7, CP5, CP3, CP1, CPz, CP2, CP4, CP6, TP8, P7, P5, P3, P1, Pz, P2, P4, P6, P8, PO7, PO5, PO3, POz, PO4, PO6, PO8, O1, Oz, O2, M1 and M2). Electrodes were mounted on a Waveguard EEG cap (Advanced Neuro Technology B.V., Enschede, The Netherlands). The electrode sites of this montage were arranged according to the international 10/10-system [56] and all EEG channels were referenced to the common average of all scalp electrodes. Forehead electrode AFz was used as ground electrode. Blue Sensor N disc electrodes (Ambu, Ballerup, Denmark) were placed at the outer canthi of both eyes and additionally below the left eye for horizontal and vertical electrooculography (HEOG and VEOG, respectively). All electrode impedances were kept below 10 kΩ.

### Preprocessing of EEG data

EEG data were preprocessed offline with the ASALAB (Version: 4.7.8) software package [57]. Preprocessing included down-sampling to 512 Hz, band-pass filtering between 0.5 and 20 Hz (24 dB/oct) and band-stop (notch) filtering of 50 Hz (24 dB/oct). Eye blinks and saccade-related artifacts were corrected with an artifact correction feature based on principle component analysis (PCA), as introduced by [58]. Further data analysis of artifact-free EEG data involved re-referencing to linked mastoids/linked ears (M1 and M2), segmentation into epochs for each stimulus type and condition, resulting in epochs from 100 ms before and 700 ms after stimulus onset for each of the four different experimental conditions including “Deviants” and “Standards” during the passive listening condition as well as “Deviants” and “Standards” during the active listening condition. All epochs were scanned for artifacts using an automated artifact detection algorithm. The automatic artifact rejection threshold in all epochs (between − 100 and 700 ms after stimulus onset) was set to ± 100 µV within a 400 ms interval (between 0 and 400 ms after stimulus onset). Epochs exceeding this threshold were discarded. All extracted epochs were baseline corrected using the interval from 100 ms before stimulus onset. Referencing, segmentation and baseline correction as well as any further analysis step (e.g., averaging) were done in EEGLAB (Version: 13.4.4b; [59]) and MATLAB (Version: R2013a, 8.1.0.604; The MathWorks Inc., Natick, MA, USA). Grand averaged ERPs were generated for all four aforementioned experimental conditions.

### ERP analysis and statistics

Detection of the time windows in which ERP amplitudes including the N1 and P3 were most pronounced was done with the built-in EEGLAB function “statcond” [60]. Averaged ERPs to deviant and standard stimuli (during both passive and active listening condition, respectively) were submitted to non-parametric paired *t*-tests with 5.000 permutations at all time points between 0 (stimulus onset) and 700 ms after stimulus onset with 62 electrode sites included. To control for multiple comparisons, the false discovery rate (FDR; for an introduction, see [61]) was used for all statistical analyses (as implemented in the EEGLAB function “FDR”) with an FDR-level of 5% (q = 0.05). By maintaining reasonable limits on the likelihood of false discoveries (i.e., it is suitable for a reasonable correction on a large number of comparisons), the FDR procedure provides a much better spatial and temporal resolution as compared to parametric *t*-tests using the “classical” Bonferroni correction (for an introduction, see [62]).

Regarding analysis of the MMN and the P3, difference waves were calculated from the averaged ERP waveforms elicited by deviant and standard pure tones in both experimental conditions (“Deviants” and “Standards” during the passive listening condition and “Deviants” and “Standards” during the active listening condition, respectively) using the Maas Univariate ERP Toolbox [63–65]. All time points in the MMN (50–150 ms) and the P3 time window (200–450 ms) as well all 62 scalp electrodes were included in the statistical tests (i.e., 3.224 and 9.610 total comparisons, respectively).

Difference waves were submitted to a repeated measures, two-tailed cluster-based permutation test based on the cluster mass statistic [66] using a family-wise alpha level of 0.05. Repeated measures *t*-tests were performed for each contrast (“Deviants” and “Standards” during the passive listening condition and “Deviants” and “Standards” during the active listening condition, respectively) using the original data and 2.500 random within-participant permutations of the data. For each permutation, all *t*-scores corresponding to uncorrected *p*-values of 0.01 or less were clustered: to this end, electrodes within a distance of approximately 5.44 cm were considered spatial neighbors and adjacent time points were considered temporal neighbors. The sum of the *t*-scores in each cluster is the “mass” of that cluster and the most extreme cluster mass in each of the 2.501 sets of tests was used to estimate the distribution of the null hypothesis (i.e., no difference between “Deviants” and “Standards” in the passive as well as “Deviants” and “Standards” in the active listening condition, respectively). More specifically, the assumption of the null hypothesis of the permutation test is that positive differences between conditions could have just as likely been negative differences and vice versa. Thus, the distribution of the null hypothesis is symmetric around a difference of 0. The permutation cluster mass percentile ranking of each cluster from the observed data was used to derive its *p*-value. The *p*-value of the cluster was assigned to each member of the cluster and *t*-scores that were not included in a cluster were given a *p*-value of 1. This permutation test analysis was used instead of the conventional testing of mean amplitude values because it provides much better spatial and temporal resolution while maintaining control of the family-wise alpha level (i.e., it corrects for a large number of comparisons). 2.500 permutations were used to estimate the distribution of the null hypothesis as it is over twice the number recommend by [67] for a family-wise alpha level of 0.01. For the cluster mass permutation test, all desired *p*-values, critical *t*-scores, and the corresponding family-wise alpha levels are reported (please see Results, “Passive tone oddball paradigm—ERPs (N1, MMN and P3)” and “Active tone oddball paradigm—ERPs (N1, MMN and P3)” sections).

### EEG source localization analysis

Neural generators of the ERPs were analyzed with the sLORETA software (University Hospital of Psychiatry, Zürich, Switzerland; [68]). Source estimations were done on single participants’ data and were restricted to the time windows showing significant differences in the ERP waveforms between the contrasted conditions (see EEG-ERP results, “Behavioral results—active tone oddball paradigm”, “Passive tone oddball paradigm—ERPs (N1, MMN and P3)”, “Active tone oddball paradigm—ERPs (N1, MMN and P3)”, “Passive and active deviants—passive and active standards ERPs” sections). In sLORETA, computations are based on a realistic head model [69] using the MNI-152 template [70], with the three-dimensional solution space restricted to cortical gray matter, as determined by the probabilistic Talairach atlas [71, 72]. The standard electrode positions of the MNI-152 template were taken from [56] as well as [73]. The intracerebral volume is partitioned in 6.239 voxels with a spatial resolution of 5 × 5 × 5 mm each. Thus, the obtained sLORETA images represent the standardized electric activity at each voxel in the neuroanatomic Montreal Neurological Institute (MNI) space as the exact magnitude of the estimated current density. Anatomical labels are reported in Brodmann areas (BA) in line with MNI space, with a correction to Talairach space [74]. For sLORETA no preregistration of individual subjects is required. The matching or co-registration of the individual EEG data with the MNI-152 template is based on the scalp-recorded electric potential distribution and computed on the basis of the cortical three-dimensional distribution of current density. Thus, the software automatically co-registers the data according to the head surface points (electrode locations provided in the electrode configuration file). As introduced by Nichols and Holmes [75], statistical non-parametric mapping (SnPM) was used to compute the standardized intracerebral current density distribution at time intervals or time points showing significant differences based on non-parametric voxel-by-voxel paired samples *t*-tests (with 5.000 permutations) on the three-dimensional sLORETA images. Statistical significance was assessed by defining critical thresholds (*t*_crit_) corrected for multiple comparisons (*p *< 0.01 and *p *< 0.05, respectively) for all tested voxels and time windows. The null hypotheses equaled the assumption that there were no differences between “Deviants” and “Standards” in both the passive and active listening condition, respectively.

Standardized current density values at each voxel have been computed in the solution space as a linear and weighted sum of the scalp electric potentials. Activation of a given voxel was based on the smoothness assumption, meaning that neighboring voxels show a highly synchronous activity [76]. Support comes from electrophysiological studies showing that electrical activity of neighboring neural populations is highly correlated [76, 77]. As proposed by [78], activated voxels exceeding *t*_crit_ were considered as being regions of cortical activation. Finally, statistical analysis resulted in a three-dimensional intracerebral current density distribution and obtained cortical regions were classified in relation to their corresponding BA [79] and normalized coordinates (Talairach and MMI, respectively).

## Results

### Behavioral results—active tone oddball paradigm

Participants’ reaction times (RTs) to deviant pure tones were between 223 and 379 ms (*M* = 285.6 ms, *SD* = 38.5). On average, participants responded to all trials with deviant pure tones with high accuracy (*M* = 74.8, *SD* = 0.7).

### Passive tone oddball paradigm—ERPs (N1, MMN and P3)

**Fig. 1:**
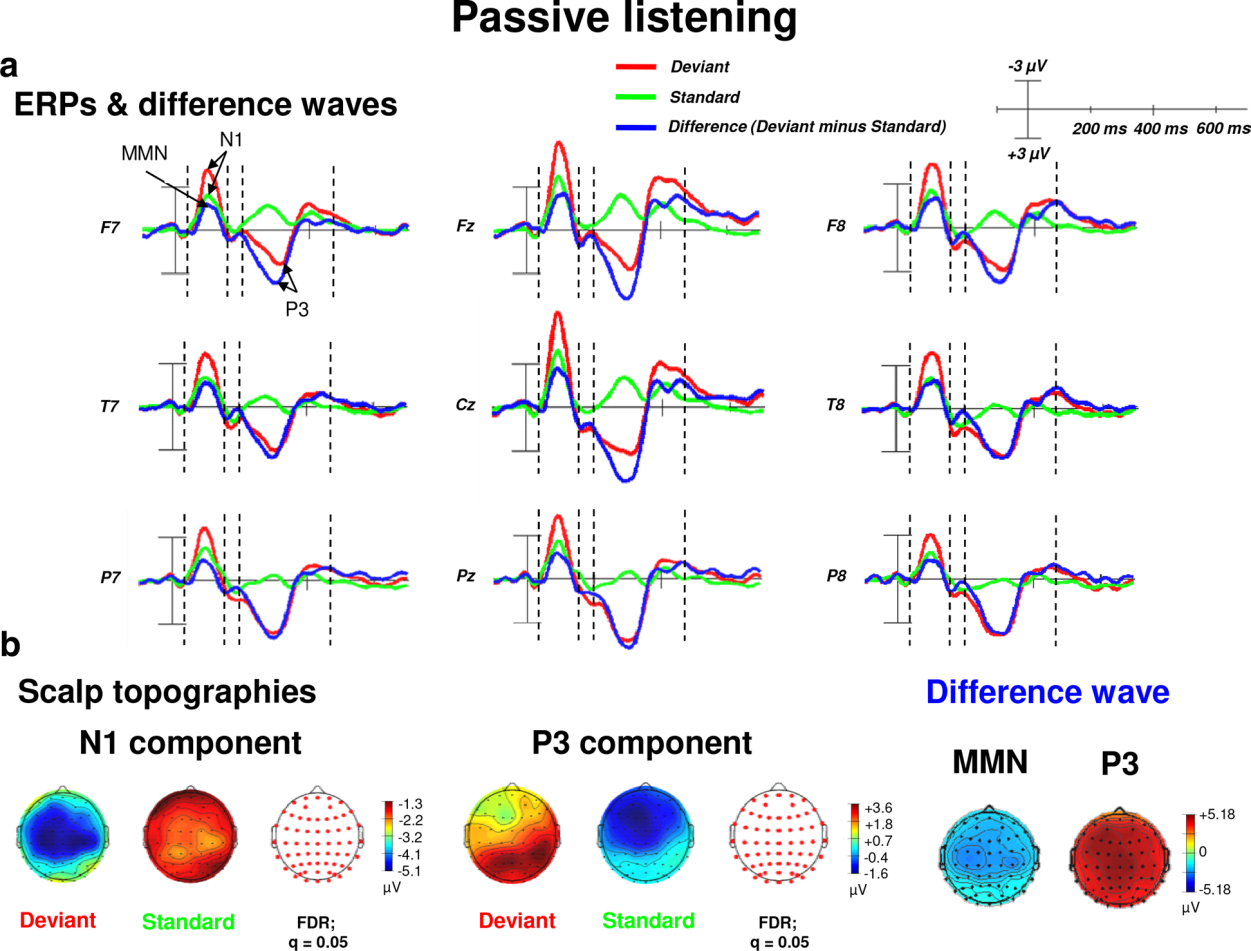
ERPs (upper panel, **a** and scalp topographic plots (lower panel, **b** for the N1 and P3 components as well as the MMN and the P3 of the difference waveforms (“Deviant” minus “ Standard”) for the passive listening condition. **a** The topographic plot shows ERP waveforms from 9 electrode sites (from frontal to posterior regions). ERP waveforms show the N1 and the P3 components with significantly higher amplitudes (more negative for the N1 and more positive for the P3) in response to deviants (pure tones with a frequency of 1000 Hz) as compared to standards (pure tones with a frequency of 500 Hz) during the passive listening condition. Furthermore, the P3 is elicited by deviants, but not in response to standards. **b** The scalp topographies of the N1 and P3 as well as the MMN and P3 (extracted difference wave) are shown in more detail. Reddish colors of the scalp indicate positive ERP values, whereas bluish colors indicate negative ERP values. In addition, the transparent EEG montage arrays (right panels) show statistically significant electrode sites as indicated by red dots (after comparison for multiple comparisons with FDR). In addition, topographic plots of the MMN and P3 peaks as difference potentials are shown (right panel). *FDR* false discovery rate, *MMN* mismatch negativity, negative is plotted up, positive is plotted downwards

Passive listening to deviant and standard pure tones elicited an N1 component as well as a P300 component. As shown in Fig. 1 and as revealed by the grand average of the ERP waveforms, N1 amplitudes were most pronounced between 67 and 129 ms (peak at about 95 ms) after stimulus onset; P3 amplitudes were most pronounced during 232–354 ms (peak at about 300 ms) post-stimulus. In addition, difference waves—subtracting ERP waveforms elicited in response to “Deviants” from “Standards”—during the passive listening condition revealed an MMN component. As shown in Fig. 1, the amplitude of the MMN overlapped with the time window of the N1 component between 66 and 128 ms after stimulus onset. As also shown in Fig. 1, amplitudes of the N1, MMN and the P3 were significantly more pronounced for deviants as compared to standards.

Regarding early time windows, the maximum number of statistically significant differences between “Deviants” and “Standards” occurred in the N1/MMN time window between 82 and 103 ms post-stimulus (desired *p* = .05, critical *t*-score = − 2.20 corresponding to a family-wise alpha-level of .05 and a Bonferroni test-wise alpha-level of .000004; see also “Passive tone oddball paradigm” section; electrodes: anterior-frontal and fronto-temporal: AF7, AF4, AF3, FT8, Fp1, Fp2, Fpz; frontal: F8, F7, F6, F5, F4, F3, F2, F1, Fz, FC6, FC5, FC4, FC3, FC2, FC1, FCz; central: C6, C4, C3, C2, C1, Cz; temporal: T8). This seems to be in line with previous findings of an ‘early’ MMN peaking at about 100 ms [32]. To ensure the validity of this interpretation, electrodes were re-referenced offline to a common average reference (CAR). According to the literature, CAR or a nose reference are recommended as these montages are known to be the best reference sites to robustly determine the MMN [80]. As expected, this procedure confirmed the characteristic polarity inversion of the extracted MMN at both mastoid electrodes sites (M1 and M2, respectively). Thus, the extracted MMN of the difference wave occurred in the averaged time window of the N1 component observed in the averaged ERP waveforms [36].

For the P3 component the maximum number of statistically significant differences between “Deviants” and “Standards” was observed between 269 and 322 ms post-stimulus (desired *p* = .05, critical *t*-score = 2.20 corresponding to a family-wise alpha-level of .05 and a Bonferroni test-wise alpha-level of .000008; see also “Passive tone oddball paradigm” section; electrodes: frontal: AF8, AF7, AF4, AF3, Fp1, Fp2, Fpz, F8, F7, F6, F5, F4, F3, F2, F1, Fz, FC6, FC5, FC4, FC3, FC2, FC1, FCz, FT8, FT7; central: C6, C5, C4, C3, C2, C1, Cz, CP6, CP5, CP4, CP3, CP2, CP1, CPz; temporal: T8, T7, TP8, TP7; parietal: P8, P7, P6, P5, P4, P3, P2, P1, Pz, PO8, PO7, PO6, PO5, PO4, PO3, POz; occipital: O2, O1, Oz).

### Active tone oddball paradigm—ERPs (N1, MMN and P3)

**Fig. 2:**
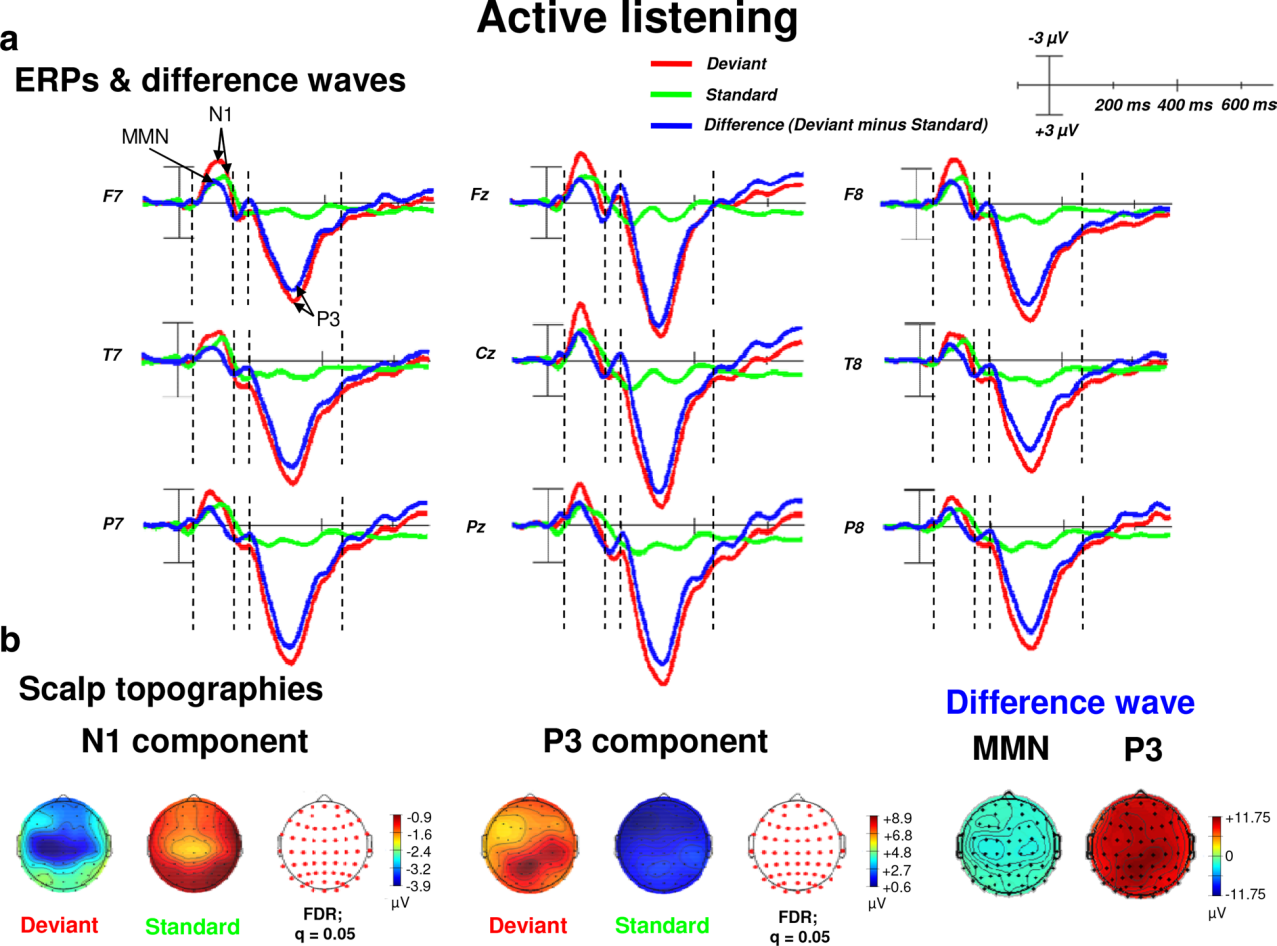
ERPs (upper panel, **a** and scalp topographic plots (lower panel, **b** for the ERP components N1 and P3 components as well as the MMN and the P3 of the difference waveforms (“Deviant” minus “Standard”) for the active listening condition. **a** The topographic head plot shows ERP waveforms from 9 electrode sites (from frontal to posterior regions). ERP waveforms show the N1 and the P3 component with significantly higher amplitudes (more negative for the N1 and more positive for the P3) in response to deviants (pure tones with a frequency of 1000 Hz) as compared to standards (pure tones with a frequency of 500 Hz) during the passive listening condition. Furthermore, the P3 is elicited by deviants, but not in response to standards. **b** The scalp topographies of the N1 and P3 as well as the MMN and P3 (extracted difference wave) are shown in more detail. Reddish colors of the scalp indicate positive ERP values, whereas bluish colors indicate negative ERP values. In addition, the transparent EEG montage arrays (right panels) show statistically significant electrode sites as indicated by red dots (after comparison for multiple comparisons with FDR). In addition, topographic plots of the MMN and P3 as difference potentials are shown (right panel). *FDR* false discovery rate, *MMN* mismatch negativity, negative is plotted up, positive is plotted downwards

Active listening to deviant and standard pure tones elicited an N1, an MMN as well as a P300 component. As shown in Fig. 2, the MMN component (obtained from the difference wave by subtracting “Deviants” from “Standards” during the active listening condition) was again overlapping with the time window of the N1 component elicited in response to “Deviants” and “Standards”. N1 amplitudes were most pronounced in the time window from 60 to 114 ms (peak: at about 90 ms), the MMN amplitude was most pronounced between 56 and 117 ms post-stimulus and the P3 amplitudes were most pronounced during 232–378 ms (peak: at about 300 ms) post-stimulus. As also shown in Fig. 2, amplitudes of the N1, MMN and the P3 were more pronounced for deviants as compared to standards, i.e., amplitudes were more negative going for the N1 and MMN and more positive going for the P3 when listening to “Deviants” as compared to “Standards”.

Regarding early time windows (N1, MMN), the maximum number of statistically significant differences between “Deviants” 3.5.2and “Standards” was observed between 83 and 95 ms post-stimulus (desired *p* = .05, critical *t*-score = − 2.21 corresponding to a family-wise alpha-level of .05 and a Bonferroni test-wise alpha-level of .000016; see also “Active tone oddball paradigm” section; electrodes: frontal: AF8, F8, F7, F6, FC6, FC5, FC4, FC3, FC2, FC1; central: C6, C5, C4, C3,C2, C1, Cz, CP6, CP4, CP3, CP2, CP1, CPz; parietal: Pz). Again (see “Passive tone oddball paradigm—ERPs (N1, MMN and P3)” section), re-referencing to CAR confirmed the characteristic polarity inversion of the extracted MMN at the left and right mastoid electrodes sites (M1 and M2, respectively).

For the P3 component the maximum number of statistically significant differences between “Deviants” and “Standards” was observed between 253 and 351 ms after stimulus onset (desired *p* = .05, critical *t*-score = 2.10 corresponding to a family-wise alpha-level of .05 and a Bonferroni test-wise alpha-level of .000008; see also “Active tone oddball paradigm”; electrodes: frontal: AF8, AF7, AF4, AF3, Fp2, Fp1, Fpz, F8, F7, F6, F5, F4, F3, F2, F1, Fz, FC6, FC5, FC4, FC3, FC2, FC1, FCz, FT8, FT7; central: C6, C5, C4, C3, Cz, CP6, CP5, CP4, CP3, CP2, CP1, CPz, C6, C5, C4, C3, C2, C1, Cz, CP4, CP3, CPz; temporal: T8, T7, TP8, TP7; parietal: P8, P7, P6, P5, P4, P3, P2, P1, Pz, PO8, PO7, PO6, PO5, PO4, PO3, POz; occipital: O2, O1, Oz).

### Passive and active deviants—passive and active standards ERPs

Contrasting ERP waveforms elicited by passive and active deviant pure tones during both oddball listening conditions revealed significant differences in ERP amplitudes between 228 and 456 ms post-stimulus onset, corresponding to the P3 component (see also “Passive and active deviants” section). In this time window, the amplitudes were more negative going during passive as compared to active listening (see Figs. 1 and 2). No amplitude differences were found in earlier time windows. Contrasting ERP waveforms elicited by standard pure tones during active versus passive listening revealed a significant difference in ERP amplitudes between 190 and 418 ms after stimulus onset (see also “Passive and active standards” section). No significant differences were found in earlier time windows corresponding to the N1 or MMN component.

### sLORETA source localization analysis

#### Passive tone oddball paradigm

**Fig. 3:**
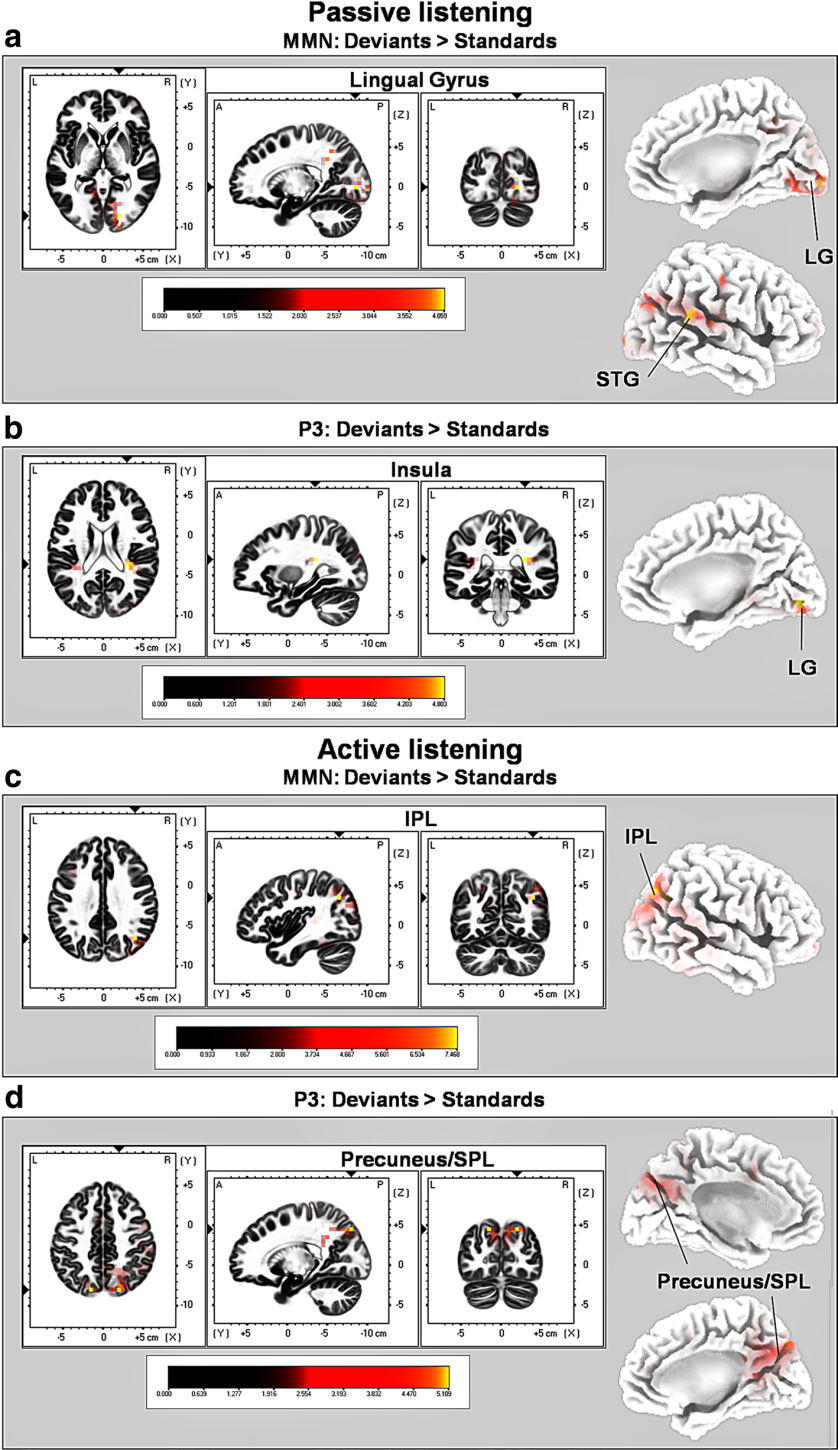
Results of the standardized low-resolution brain electrotomography (sLORETA) source localization analysis in the ‘passive’ and ‘active’ pure tone oddball paradigm. Images have been obtained after statistical non-parametric mapping (SnPM) and co-registration to the stereotaxic Talairach space based on the Co-Planar Stereotaxic Atlas of the Human Brain [72] and the probabilistic MNI-152 template [70]. Activated voxels are indicated by yellowish and reddish colors [after correction for multiple comparisons (*p *< 0.01 and *p *< 0.05, respectively)]. **a** In the averaged time windows of the ‘unattended’ MMN component (80–123 ms), the peak of highest cortical activity has been found in the right LG (BAs 17/18/19) and right STG (BAs 13/22/39/42). **b** In the averaged time windows of the ‘attended’ MMN component (83–95 ms), the peak of highest cortical activity has been found in the right IPL (BAs 7/39/40). **c** In the averaged time windows of the ‘unattended’ P3 component (269–322 ms), the peak of highest cortical activity has been found in both insulae bilaterally (BA 13) and the right LG (BA 18). **d** In the averaged time windows of the ‘attended’ P3 component (253–351 ms), the peak of highest cortical activity has been found in the precuneus/SPL bilaterally (BAs 7/19/23/21). *L* left, *R* right, *LG* lingual gyrus, *STG* superior temporal gyrus, *IPL* inferior parietal lobule, *SPL* superior parietal lobule, *MNI* Montreal Neurological Institute, *X, Y, Z* corresponding MNI coordinates, *BA* Brodmann area

As shown in Fig. 3, contrasting deviant against standard pure tones with sLORETA during the passive oddball listening condition (contrast: “Deviants” >“Standards”) revealed significant activations in the lingual gyri (bilaterally) (BAs 17/18/19; *t*-score 4.06; *p *< 0.01) as well as in the right superior temporal gyrus (STG; BAs 13/22/39/42; *t*-score 3.97; *p *< 0.05) between 82 and 103 ms after stimulus onset, which corresponds with the ERP analysis time window in which N1 and MMN amplitude differences were most pronounced, see “Passive tone oddball paradigm—ERPs (N1, MMN and P3)").

In addition, between 269 and 322 ms (P3 component) significant electrocortical activations included the insular cortex bilaterally (BA 13; *t*-score 4.80; *p *< 0.01) and the right lingual gyrus (LG; BA 18; *t*-score 4.78; *p *< 0.01), see Fig. 3. For a complete overview of all retrieved statistically significant results including all anatomical regions and activated voxels, see Tables 1 and 2.

**Table 1:** sLORETA results from the contrast: “Deviant” versus “Standard” (1000 vs. 500 Hz pure tones) in the N1/MMN time window from 80 to 123 ms post stimulus-onset during passive listening

Brain region				Coordinates (X, Y, Z)						*t*-value		No. of voxels
Structure	BA	Hemisphere	Lobe	Talairach (max.)			MNI (max.)			Max.	Min.	
Lingual gyrus	*17*, 18, 19	R/L	Occipital	20	− 82	4	20	− 85	0	4.06**	3.30*	30
Superior Temporal Gyrus (STG)	13, *22*, 39, 42	R	Temporal	64	38	20	65	− 40	20	3.97*	3.29*	9

Talairach/MNI coordinates and *t*-values are referred to the peak activity in each brain region. Italic numbers indicate maximal brain electrical activity in the corresponding BA. Only clusters of size ≥ 9 voxels are reported

**p < 0.01, *p < 0.05, *L* left, *R* right, *BA* Brodmann area, *MNI* Montreal Neurological Institute

**Table 2:** sLORETA results from the contrast: “Deviant” versus “Standard” (1000 vs. 500 Hz pure tones) in the N1/MMN time window from 83 to 95 ms post stimulus-onset during active listening

Brain region				Coordinates (X, Y, Z)						*t*-value		No. of voxels
Structure	BA	Hemisphere	Lobe	Talairach (max.)			MNI (max.)			Max.	Min.	
Inferior Parietal Lobule (IPL)	7, *39*, 40	R	Parietal/Temporal	45	− 66	40	45	− 70	40	8.12**	4.66**	10

Talairach/MNI coordinates and *t*-values are referred to the peak activity in each brain region. Italic numbers indicate maximal brain electrical activity in the corresponding BA. Only clusters of size ≥ 9 voxels are reported

**p < 0.01, *L* left, *R* right, *BA* Brodmann area, *MNI* Montreal Neurological Institute

#### Active tone oddball paradigm

Contrasting deviant against standard pure tones in the active oddball listening condition (contrast: “Deviants” > “Standards”) with sLORETA revealed significant activation in the right inferior parietal lobule (IPL; BAs 7/39/40; *t*-score = 8.12; *p *< 0.01) between 83 and 95 ms after stimulus onset and hence, in the time window of the N1 and MMN amplitude (see ERP results in 3.3). Furthermore, significant activations were found in the precuneus bilaterally (BAs 7/19/23/31; *t*-score 5.11; *p *< 0.01), the cingulate cortices bilaterally (BA 31; *t*-score 4.86; *p *> 0.01), the superior temporal gyri bilaterally (STG; BAs 13/22/39/41; *t*-score 4.79; *p *< 0.01), the left and right precentral gyri (BAs 4/6; *t*-score 4.81; *p *< 0.01), the left and right postcentral gyri (BAs 2/3/7/40; *t*-score 4.64; *p *< 0.01), the right posterior cingulate cortex (BAs 23/30/31; *t*-score 4.59, *p *< 0.01) and the right intraparietal lobe (IPL, BAs 39/40; *t*-score 4.04; *p *< 0.01) in the time window between 253 and 351 ms (overlapping with the time window of the P3 component), see Fig. 3.

For a complete overview of all retrieved statistically significant results including all anatomical regions and activated voxels, see Tables 3 and 4.

**Table 3:** sLORETA results from the contrast: “Deviant” versus “Standard” (1000 vs. 500 Hz pure tones) in the P3 time window from 269 to 322 ms post stimulus-onset during passive listening

Brain region				Coordinates (X, Y, Z)						*t*-value		No. of voxels
Structure	BA	Hemisphere	Lobe	Talairach (max.)			MNI (max.)			Max.	Min.	
Insula	*13*	R/L	Sub-lobar	30	− 33	20	30	− 35	20	4.80**	3.59*	11
Lingual Gyrus	*18*	R	Occipital	10	− 78	0	10	− 80	− 5	4.78**	3.59*	11

Talairach/MNI coordinates and *t*-values are referred to the peak activity in each brain region. Italic numbers indicate maximal brain electrical activity in the corresponding BA. Only clusters of size ≥ 9 voxels are reported

**p < 0.01, *p < 0.05, *L* left, *R* right, *BA* Brodmann area, *MNI* Montreal Neurological Institute

**Table 4:** sLORETA results from the contrast: “Deviant” versus “Standard” (1000 vs. 500 Hz pure tones) in the P3 time window from 253 to 351 ms post stimulus-onset during active listening

Brain region				Coordinates (X, Y, Z)						*t*-value		No. of voxels
Structure	BA	Hemisphere	Lobe	Talairach (max.)			MNI (max.)			Max.	Min.	
Precuneus	*7*, 19, 23, 31	R/L	Parietal/Occipital	20	− 75	45	20	− 80	45	5.11**	4.03**	132
Cingulate Gyrus	*31*	R/L	Limbic	15	− 42	25	15	− 45	25	4.86**	4.06**	20
Precentral Gyrus	4, *6*	R	Frontal	45	− 8	37	45	− 10	40	4.81**	4.08**	13
Superior Temporal Gyrus (STG)	13, 22, 39, *41*	R/L	Temporal	− 50	− 28	15	− 50	− 30	15	4.79**	4.04**	9
Postcentral Gyrus	2, 3, 7, *40*	R/L	Parietal	− 50	− 24	15	− 50	− 25	15	4.64**	4.03**	11
Cuneus	7, 18, *19*, 30	R/L	Occipital	− 10	− 76	36	− 10	− 80	35	4.62**	4.04**	35
Posterior Cingulate	23, 30, *31*	R	Limbic	10	− 53	16	10	− 55	15	4.59**	4.05**	13
Inferior Parietal Lobule (IPL)	39, *40*	R	Parietal	35	− 47	39	35	− 50	40	4.45**	4.04**	22

Talairach/MNI coordinates and *t*-values are referred to the peak activity in each brain region. Italic numbers indicate maximal brain electrical activity in the corresponding BA. Only clusters of size ≥ 9 voxels are reported

**p < 0.01, *L* left, *R* right, *BA* Brodmann area, *MNI* Montreal Neurological Institute

#### Passive and active deviants

Contrasting “Deviants” elicited during active listening against “Deviants” elicited during passive listening (i.e., Deviants active > Deviants passive) revealed significant differences in activation in the right middle frontal gyrus (MTG; BAs 6/8/9/10/46; *t*-score 5.24, *p *< 0.01), the left precuneus (BAs 7/19; *t*-score 5.06, *p *< 0.01), the right inferior frontal gyrus (IFG; BAs 9/13/45; *t*-score 4.86, *p *< 0.01), the left postcentral gyrus (BAs 2/5/7/40; *t*-score 4.80, *p *< 0.01), the left superior parietal lobule (SPL; BA 7; *t*-score 4.77, *p *< 0.01), the right precentral gyrus (BAs 6/9; *t*-score 4.36, *p *< 0.01), and the right inferior parietal lobule (IPL; BA 40; *t*-score 4.27, *p *< 0.01) in time window between 244 and 343 ms, corresponding to the time window in which amplitudes of the P3 component were most pronounced during passive and active listening conditions (see “Active tone oddball paradigm—ERPs (N1, MMN and P3)”, “Passive and active deviants—passive and active standards ERPs” sections), see Table 5.

**Table 5:** sLORETA results from the contrast: “Deviant” during active listening versus “Deviant” during passive listening in the time window between 228 and 456 ms after stimulus onset

Brain region				Coordinates (X, Y, Z)						*t*-value		No. of voxels
Structure	BA	Hemisphere	Lobe	Talairach (max.)			MNI (max.)			max.	min.	
Middle frontal gyrus	6, 8, *9*, 10, 46	R	Frontal	54	12	36	55	10	40	5.24**	4.00**	27
Precuneus	*7*, 19	L	Parietal	− 10	− 55	63	− 10	− 60	65	5.06**	3.81*	47
Inferior frontal gyrus	*9*, 13, 45	R/L	Frontal	54	11	32	55	10	35	4.86**	3.81*	16
Superior parietal lobule	*7*	R/L	Parietal	− 15	− 51	58	− 15	− 55	60	4.77**	3.81*	11
Precentral gyrus	6, *9*	R	Frontal	45	21	36	45	20	40	4.42**	3.81*	9
Inferior parietal lobule	*40*	R	Parietal	50	− 37	43	50	− 40	45	4.27**	3.92**	12

Talairach/MNI coordinates and *t*-values are referred to the peak activity in each brain region. Itlaic numbers indicate maximal brain electrical activity in the corresponding BA. Only clusters of size ≥ 9 voxels are reported

**p < 0.01, *p < 0.05, *L* left, *R* right, *BA* Brodmann area, *MNI* Montreal Neurological Institute

#### Passive and active standards

Contrasting standards during both listening conditions (contrast: “Standards” active listening condition > ”Standards” passive listening condition) revealed significant activation in the right middle frontal gyrus (BAs 10/11; 40, 53, − 11; *t*-score 4.39; *p *< 0.05) in the time window between 190 and 418 ms. As this contrast revealed only three significant voxels, no table is provided.

## Discussion

The present study examined the spatio-temporal dynamics of auditory deviance and target detection in the auditory oddball paradigm by combining the advantages of the EEG–ERP methodology and the sLORETA source localization technique within the same experiment and subjects (i.e., within subject design). The design included both, passive as well as active listening conditions to specify and contrast the neural mechanisms underlying active and passive deviance and target detection. To this end, participants were instructed to listen (1) passively to pure tones without giving an overt behavioral response (experimental block 1) and (2) to listen to pure tones while being engaged in an active task (experimental block 2) which afforded to distinguish between the two presented pure tones by responding to the deviants by giving an overt behavioral response (button press).

### Time course of passive and active deviance and target detection

Passive and active listening elicited an N1 and P3 component and additionally an MMN as difference potential when deviants were contrasted against standards. Amplitudes of the MMN which temporally overlapped with the amplitudes of the N1 were significantly more negative for deviant as compared to standard pure tones during passive and active listening conditions. This modulation pattern is in line with those reported in previous ERP studies using comparable auditory oddball paradigms with pure tones (e.g., see [81, 82]). Comparisons between deviants or standards during active versus passive listening (see “Passive and active deviants— passive and active standards ERPs”, “Passive and active deviants” and Passive and active standards” sections) revealed no significant amplitude differences in the time window of the N1/MMN when passive and active listening conditions were compared against each other. Thus, voluntary guidance of selective attention to deviants may not facilitate deviance detection in early time windows of cortical stimulus processing (N1/MMN) beyond passive listening. This finding supports the assumption of particularly the MMN reflecting pre-attentive sensory stimulus discrimination [38] and automatic (involuntarily) auditory change detection [39, 40], i.e., processes that cannot be influenced by task-related attentive processes. Regarding the overlap between amplitudes of the N1 and the MMN additional explanations for MMN modulation during auditory deviance processing have been proposed: as shown in Figs. 1 and 2, the time window of the N1 significantly overlapped with the MMN. In addition, N1 amplitudes were significantly reduced to standards during passive and active listening conditions. This may be explained by the observation that neurons reacting to standards only show a reduced electrical activity due to repeated stimulus presentation leading to habituation of these particular neurons. In contrast, neurons that fire in response to deviants show a much higher electrical activity. According to the literature, this phenomenon might be explained by the so-called refractoriness of certain neurons and thus by their selective sensitivity to different frequencies [36, 83, 84]. Hence, due to the amplitude overlap of the MMN and the N1, MMN modulation could also result from neural adaption in the auditory cortex [85]. However, whether this mechanism actually underlies N1/MMN overlap has not been sufficiently clarified yet and requires further neurophysiological testing.

### Neural sources of passive and active deviance and target detection

#### Early time windows (N1/MMN)

When passive listening to “Deviants” vs. “Standards” was compared, source localization with sLORETA revealed activation in the left and right occipital cortex as well as in the right superior temporal gyrus (STG; BA 22) in the N1/MMN time window. Activation of the right STG included the auditory cortex (BA 42) and multisensory association areas (BAs 39/22). This is well in line with the idea of bottom-up and stimulus-driven deviance detection. During active processing of “Deviants” vs. “Standards” the largest voxel cluster was located in the right inferior parietal lobule (IPL; BAs 39/40). The right IPL is part of the ventral attention network (VAN) and plays an important role in visuo-spatial attention and attentive monitoring of stimuli for goal-directed eye or limb movements [86]. Thus, during active listening IPL activation during early stages of deviance processing (i.e., in the N1/MMN time window) may be a consequence of anticipatory control of attention in order to maintain current task goals (i.e., the voluntary selection of deviant stimuli). Altogether, this suggests that during passive and active listening early stages of deviance processing may be modulated by different brain regions and neural processes although, at a cortical level, with respect to ERPs, N1 and MMN amplitude modulation did not differ significantly between passive and active listening conditions.

#### Late time windows (P3)

For the time window corresponding to the P3 component the contrast “Deviants” > ”Standards” revealed activation of the right and left insula during passive listening. Activation of the left and the right insula in the P3 window during passive listening is in line with previous studies reporting involvement of the insula (in particular the right insula) in auditory processing [87] and target detection (e.g., see [88]). More specifically, the insula is part of the VAN and also part of the so-called *salience network* (SN) [18, 19] that marks stimulus events as salient for additional processing and mediates activation of and between brain networks involved in bottom-up and top-down controlled attention. Crucially, during active listening, P3 modulation elicited by deviants as compared to standards was associated with activation in a distributed network including the precuneus and surrounding areas in the superior parietal lobule (SPL; BA 7), the posterior cingulate cortex (PPC; BAs 23/31) as well as motor-related areas. Most of the aforementioned brain regions form part of the dorsal attention networks (DAN). The present results therefore suggest that activation of brain regions belonging to the DAN network may occur only during active listening and during later time windows (P3) associated with voluntary guided target detection. Interestingly, the largest voxel cluster (size: 132 voxels) was located within the precuneus/SPL bilaterally. The precuneus is a cortical structure located in the superior parietal cortex. It is one of the core structures of the DAN [4, 14] and associated with voluntary attentional switching [89], but also modulated by saliency [90]. Activation of the precuneus/SPL (e.g., BA 7) was also observed in the contrast comparing processing of deviants during active > passive listening; again in the time P3 time window. In addition, P3 modulation was significantly larger for “Deviants” during active as compared to passive listening. Activation of the precuneus/SPL in the P3 time window during active listening may therefore indeed indicate the increase in attentional demands from passive to active, attentive and thus, voluntary and top-down controlled target detection.

Taken together, the sLORETA source localization analysis support the hypothesis that unattended (passive) as well as attended deviance and target detection elicit cortical activations in spatially distributed brain regions belonging to different brain networks including the VAN, DAN and SN.

### A neurophysiolological model of passive and active auditory deviance and target detection

**Fig. 4:**
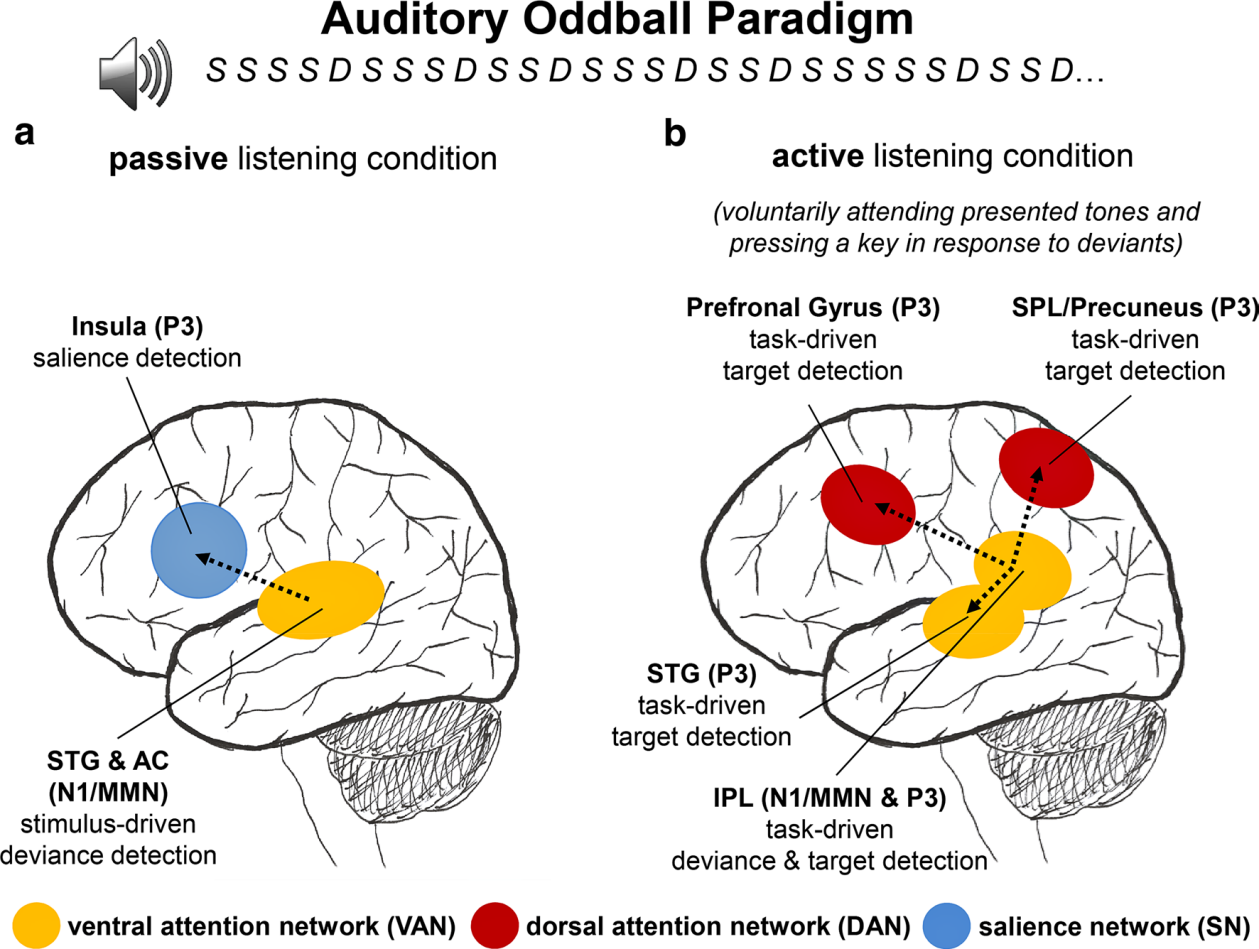
Overview of a proposed model based on the obtained ERP and sLORETA results (for an explanation, see "A neurophysiolological model of passive and active auditory deviance and target detection" section). *AC* auditory cortex, *IPL* inferior parietal lobule, *MMN* mismatch negativity, *STG* superior temporal gyrus, *SPL* superior parietal lobule

As illustrated in Fig. 4, based on the results of the present study a neurophysiological model of passive and active auditory deviance and target detection can be proposed that may act as a guide for future research. As shown in Fig. 4, this model illustrates that passive and active auditory deviance detection in the auditory oddball paradigm are associated with activation of brain regions belonging to at least two different brain networks: these include on the one hand auditory processing regions in the STG (e.g., BA 22) and the insula (BA 13) as key region of the VAN and SN involved in passive auditory deviance detection, and on the other hand, parietal and frontal brain regions as key regions of the VAN and particularly of the DAN involved in task-related auditory deviance and target detection [18, 91]. Activation of the STG and insula during passive listening is in close agreement with previously conducted neuroimaging studies combining fMRI with multi-channel EEG recordings during an auditory oddball paradigm and a passive listening task [10, 12]. Results of these studies obtained from fMRI indicated comparable cortical activations in the right STG and the right superior temporal plane (BAs 41/42) and right anterior insula during passive listening of auditory deviant stimuli. Also, going beyond previous research, the present results suggest that during passive listening activation of the VAN and the SN in particular may occur at later stages of stimulus selection, i.e., when deviants in contrast to standards are selected for further processing and associated with the elicitation of a P3 component. Earlier processing stages associated with automatic deviance detection as reflected by N1/MMN modulation on the contrary seem to be related specifically with activation of sensory brain regions belonging to the VAN (superior temporal gyrus) as well as with visual cortical activation. Although brain regions in the visual cortex, such as the lateral occipital cortex are typically associated with the processing of visual information (e.g., visual objects) in visual spatial attention tasks [21–23], more recent functional imaging studies [9, 92] showed in line with our findings that specific regions in the visual cortex (such as the LOC or the lingual gyrus in the medial visual cortex) may be activated during the processing of salient acoustic stimuli [24] and as suggested by our study this may occur even or specifically if no task is at hand. Moreover, in contrast to passive listening, during active listening auditory processing may be fully taken over by the brain’s attention networks including activation of brain regions belonging to the VAN during deviance detection in the N1/MMN time window and of the DAN during target detection in the P3 time window.

### Limitations and future outlook

Although the results of the present study including the proposed model support a number of the hypotheses tested there are limitations that must be taken with caution. A major disadvantage of the present study may be the small sample size. However, effect sizes calculated for the *t*-tests reported under 3.2–3.5 revealed at least moderate effects (Cohen’s *d* ≥ |0.6|) resulting in a post hoc power estimation of at least 0.6. Nevertheless, due to the small sample size the generalizability of the reported effects and the proposed model may be limited (see Fig. 4).

Another confound in the present study might be the temporal overlap of the N1 and MMN component. This phenomenon may be observed when the perceptual difference between “Deviants” and “Standards” is particularly prominent in the frequency domain [93]. Regarding the question which and how many brain networks may be activated during passive versus active deviance and target detection further research may unravel the functional connectivity between the hypothesized networks. The present results suggest that besides the VAN and DAN, the SN may indeed play an important role in auditory deviance and target detection during passive listening. In contrast to the VAN, the SN is believed to be involved in the detection of stimulus saliency, expectancy and automatic selection of an adaptive and suitable (behavioral) response [18]. The core structure of the SN consists of the dorsal part of the ACC (dACC; BAs 24/32/33), subcortical and limbic structures (e.g. amygdala), as well as both insulae bilaterally [91]. Given the high degree of functional and anatomical overlap between the SN and the VAN, some researchers see both networks as parts of the same system [94]. However, given that according to meta-analytic findings the VAN should be more active when stimuli are task-relevant which was confirmed in this study and the fact that, in this study activation of the insula was not found during active listening, the present results agree with the notion to conceptualize the SN and VAN as two distinct networks [95, 96] with distinct roles during auditory processing.

Yet, another restriction that needs to be mentioned is related to the mathematical algorithms implemented in sLORETA. These algorithms are mainly based on non-parametric voxel-by-voxel comparisons for why sLORETA results—like conventional fMRI results—should not be interpreted causally. To overcome some of these methodological limitations, the application of repetitive transcranial magnetic stimulation (rTMS) may offer a non-invasive and thus elegant way to selectively inhibit or facilitate cortical activity in superficial brain regions (e.g., IPL or SPL/precuneus) and even in deeper cortical structures such as the insula [97] by applying fast trains of electromagnetic pulses [98]. Hence, in future studies, rTMS would offer potential prove for the neurophysiological model derived from the present data regarding passive as well as active auditory deviance and target detection.

## Conclusion

In summary, the present study investigated the temporal and spatial dynamics of auditory deviance and target detection in an auditory oddball paradigm by combining EEG–ERP and sLORETA methods. Despite abundant previous research investigating either the time course or the neural sources and brain structures of auditory processing in the auditory oddball paradigm the present study is one of the few studies so far that combined analysis of ERPs with sLORETA source imaging during passive and active listening conditions in a within subject design in an attempt to explore when and where in the brain auditory deviance and target detection takes place during passive and active listening conditions. The results of the present study as well as the neurophysiological model derived from the current findings may be tentative due to the small sample size but may bolster future studies validating the suggested temporal activation pattern in larger samples of participants.
